# Wearable Sensor Data to Track Subject-Specific Movement Patterns Related to Clinical Outcomes Using a Machine Learning Approach

**DOI:** 10.3390/s18092828

**Published:** 2018-08-27

**Authors:** Dylan Kobsar, Reed Ferber

**Affiliations:** 1 Faculty of Kinesiology, University of Calgary, 2500 University Dr NW, Calgary, AB T2N 1N4, Canada; rferber@ucalgary.ca; 2 Running Injury Clinic, Calgary, AB T2N 1N4, Canada; 3 Faculty of Nursing, University of Calgary, Calgary, AB T2N 1N4, Canada

**Keywords:** accelerometer, machine learning, pattern recognition, sensors, gait, clinical, knee, osteoarthritis

## Abstract

Wearable sensors can provide detailed information on human movement but the clinical impact of this information remains limited. We propose a machine learning approach, using wearable sensor data, to identify subject-specific changes in gait patterns related to improvements in clinical outcomes. Eight patients with knee osteoarthritis (OA) completed two gait trials before and one following an exercise intervention. Wearable sensor data (e.g., 3-dimensional (3D) linear accelerations) were collected from a sensor located near the lower back, lateral thigh and lateral shank during level treadmill walking at a preferred speed. Wearable sensor data from the 2 pre-intervention gait trials were used to define each individual’s typical movement pattern using a one-class support vector machine (OCSVM). The percentage of strides defined as outliers, based on the pre-intervention gait data and the OCSVM, were used to define the overall change in an individual’s movement pattern. The correlation between the change in movement patterns following the intervention (i.e., percentage of outliers) and improvement in self-reported clinical outcomes (e.g., pain and function) was assessed using a Spearman rank correlation. The number of outliers observed post-intervention exhibited a large association (ρ = 0.78) with improvements in self-reported clinical outcomes. These findings demonstrate a proof-of-concept and a novel methodological approach for integrating machine learning and wearable sensor data. This approach provides an objective and evidence-informed way to understand clinically important changes in human movement patterns in response to exercise therapy.

## 1. Introduction

As wearable sensors become more and more ubiquitous in today’s world, so does their use in human movement analysis. Given their size, affordability and ease of use, wearable inertial sensors can provide a clinically accessible alternative to more expensive conventional three-dimensional (3D) motion capture systems [1]. A further advantage of these devices is that they can be placed at variety of body locations (e.g., wrist, torso, lower limbs, etc.) and collect data continuously over many strides both in and outside of a laboratory or clinical setting [2]. However, a fundamental problem in collecting data from multiple sensors over long periods of time is that datasets quickly become exceedingly large, complex, and, most importantly, clinically uninterpretable. To counter this problem, many clinical investigations have examined simple wearable sensor outputs such as gait speed, step times and other discrete variables [3,4,5]. Nevertheless, there remains a vast amount of data being created by today’s wearable sensors that goes unanalyzed and may itself hold the key to answering important clinical questions [6]. Therefore, there is a need to develop more sophisticated and complex methods to extract, process and present this data in clinically meaningful ways [6,7,8,9].

Perhaps the most promising way to utilize the vast amounts of data generated by modern wearable sensors is the field of machine learning [6]. Machine learning involves the integration of statistics and computer science to identify patterns in large datasets [10]. These pattern recognition tools provide the opportunity to quickly process and compare large wearable sensor datasets between or within different clinical populations or subgroups [11,12,13,14,15]. While group-based models can be effective in identifying clinically relevant differences in gait between individuals, there remain a number of important limitations. Most notably, group-based models require the curation of datasets that contain a large number of subjects to externally validate a model [16]. In the world of clinical gait biomechanics, even with the support of wearable sensors, recruiting and testing large numbers of patients can be difficult or impractical. Moreover, with the large amount of heterogeneity in some clinical populations (e.g., osteoarthritis; OA [17]), this becomes even more difficult.

Alternatively, subject-specific models can be used to identify gait patterns and track changes within a single patient. Used in conjunction with wearable sensors collecting data over hundreds or thousands of individual gait cycles, these subject-specific models have demonstrated the ability to outperform group-based models in identifying clinically relevant changes in gait [18,19]. A unique approach to this model is the application of a one-class classification algorithm, wherein the machine learning algorithm attempts to define the multivariate properties or boundaries for a typical observation in the dataset [20]. When applied to a gait pattern, this approach can define the current state of a subject’s gait pattern and, in turn, identify changes or outliers from the original pattern. Moreover, this method can do this without the need for additional training data from conditions that may be currently unknown or yet to exist (e.g., individual data on future injury states or rehabilitation outcomes). Research has examined the ability of similar, subject-specific one-class models to identify typical gait patterns in cattle [21] and, more notably, deviations from baseline human gait patterns when perturbed by a knee brace [22]. However, to our knowledge no research has examined the opposite proposition of identifying potentially advantageous deviations from a baseline gait pattern over the course of an intervention in a clinical population.

Therefore, the objective of this research was to establish a proof-of-concept of a subject-specific one-class model’s ability to identify clinically relevant changes in gait patterns over the course of rehabilitation. Specifically, our research question was: are changes in gait patterns correlated with clinical improvements following a 6-week exercise intervention in knee OA patients? It was hypothesized that patients who benefited most from the exercise intervention (i.e., improvements in self-reported pain, function, etc.) would also demonstrate the greatest changes in their gait patterns (i.e., increased percentage of outlier gait cycles) following the intervention, as assessed by a Spearman’s rank correlation (α < 0.05).

## 2. Methods

### 2.1. Subjects

A subset of 8 knee OA patients (Sex: 4F/4M, Age: 58 (5) years, Body Mass Index: 25.3 (4.8) kg/m^2^, walking speed: 1.1 (0.15) m/s) were analyzed from a larger exercise intervention [11]. These patients were selected for the current analysis as they completed two baseline gait trials before the intervention, as well as one gait trial post-intervention. All participants were required to be radiographically diagnosed with knee OA and able to walk without assistive devices. For additional inclusion and exclusion criteria see Kobsar et al., 2017 [11]. This study was approved by the Conjoint Health Research Ethics Board at the University of Calgary (E-22417: Approved 14 June 2014) and all participants provided written, informed consent prior to participating.

### 2.2. Protocol

**Figure 1:**
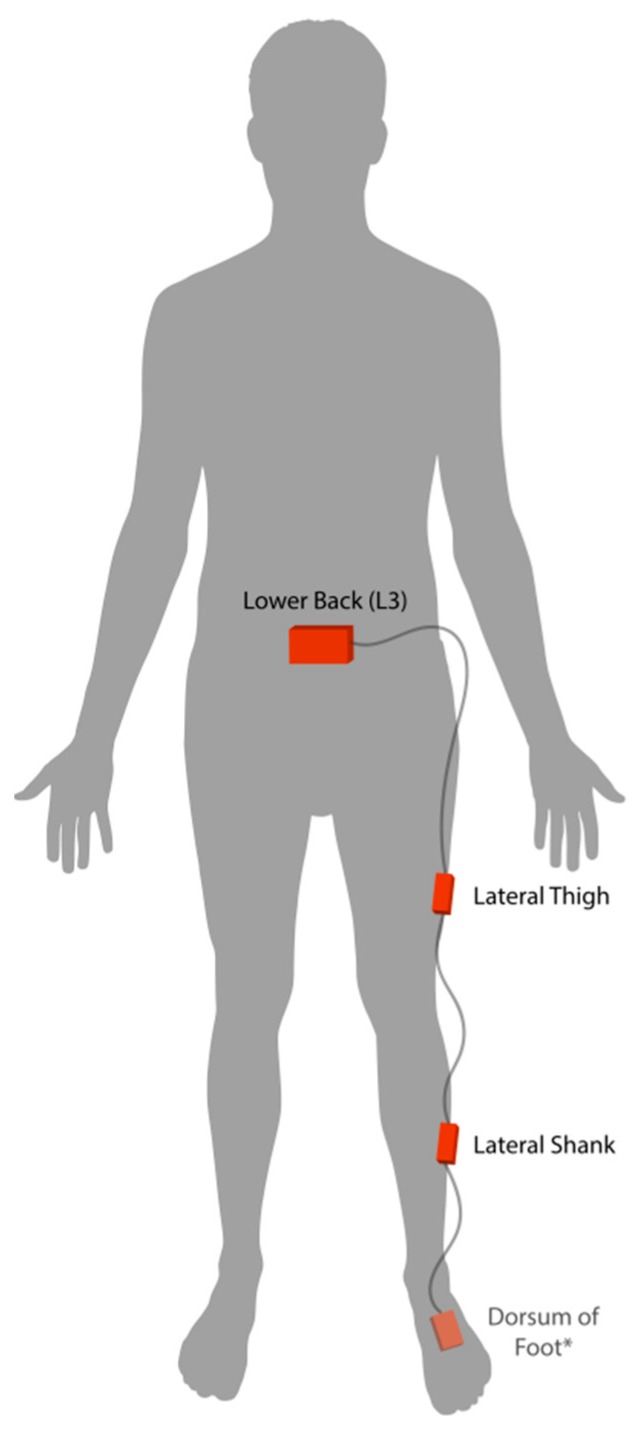
Placement of inertial sensors on the most affected side of knee osteoarthritis patients. * Sensor on the dorsum of the foot was only used for event detection and gait cycle segmentation.

Participants completed two baseline gait trials on different days, within one week of beginning the exercise intervention. In each session, participants wore four wearable inertial sensors (iNEMO inertial module, STmicroelectronics, Geneva, Switzerland) securely fastened to their lower back (approximately the L3 vertebrae), lateral thigh, lateral shank and dorsum of the foot on the most affected leg. See Figure 1. In order to safeguard against potentially high impact accelerations at the foot, the highest accelerometer and gyroscope range setting (acceleration range ±16 g, gyroscope range ±2000°/s, sampling rate 100 Hz) was used for the foot sensor. Further, the same range settings were selected across all remaining sensors to maintain data consistency. While all four sensors collected both linear acceleration and angular velocity data, only linear acceleration data from the three most proximal sensors (i.e., lower back, thigh and shank) were used for modeling the subject-specific gait patterns. The fourth sensor on the dorsum of the foot was used solely for gait event detection, to be discussed in the following section. 

All participants walked on a level treadmill (Bertec, Columbus, OH, USA) for a short acclimatization period at a self-selected pace, before 2.5 min of data were collected. A trial length of 2.5 min was used to obtain a minimum of 100 gait cycles per session, as this has been a recommended for similar machine learning analyses [23]. The same protocol and self-selected pace were used during the post-intervention gait trial. Participants also completed a Knee injury and Osteoarthritis Outcome Score (KOOS) before and after the intervention to assess changes, if any, in self-reported pain, symptoms, function in daily living and knee related quality of life following the intervention [24]. The exercise intervention itself was a 6-week therapist-directed, hip-focused muscle strengthening intervention detailed in Kobsar et al., 2017 [11]. 

### 2.3. Data Analysis

#### 2.3.1. Pre-Processing

Linear acceleration data underwent a static attitude correction to align each sensor with the global vertical and horizontal planes [25,26]. Following this correction, all 3D linear acceleration and angular velocity data were filtered with a 10 Hz low-pass 4th order recursive Butterworth filter. The angular velocity data of the foot sensor was used to determine gait events (e.g., initial contact and toe-off) in a manner previously validated [26,27,28]. Specifically, initial contact and toe-off events were determined as the zero-crossing preceding stance and the negative peak following stance in the angular velocity signals about mediolateral axis, respectively. These gait events allowed for gait cycle segmentation and time-normalization of all sensor data (e.g., 60 points for stance; 40 points for swing). Three-dimensional linear accelerations were concatenated within each sensor (i.e., 3 axes × 100 data points combined to a 1 × 300 vector) and across all 3 sensors (i.e., 1 × 300 vectors from back, thigh and shank sensors combined) to form a 1 × 900 vector which defined the overall movement pattern for each individual gait cycle. Finally, the linear acceleration data from each gait trial were stored in an m × 900 matrix, where “m” equals the number of gait cycles recorded during the 2.5 min of data collection. The average number of gait cycles per session was 135 (10). 

#### 2.3.2. Data Reduction and Feature Selection

Prior to computing the boundaries of the subject-specific one-class model, the 900 point vectors defining each gait cycle were reduced to a set of principal components (PCs). To do so, data from both baseline gait trials were combined, resulting in an average of 270 gait cycles collected over 5 min of walking data. These data were then standardized to a mean of 0 and a standard deviation of 1, before being transformed into linearly uncorrelated PCs using a principal component analysis [11,29]. The PCs that explained at least 95% of the total variance in the original data were selected as features for the algorithm [30]. Therefore, the scores on these PCs across all baseline gait cycles were used as the features to define the overall gait pattern of a subject. Given that this was a subject-specific model, a total of 8 principal component analyses were conducted on baseline data (i.e., one for each subject). Therefore, each patient had their own unique set of gait features to be used in modeling their gait pattern. Post-intervention data were reduced and features (i.e., PC scores) were computed in the same manner as the baseline. This procedure involved utilizing the data reduction outputs generated from the baseline data (i.e., mean, standard deviation and PC loading coefficients) to ensure the post-intervention PC scores were appropriately aligned with their corresponding baseline data.

#### 2.3.3. Defining Subject-Specific One-Class Models

Subject-specific, one-class models were defined using baseline gait features (i.e., reduced PC scores) in conjunction with a one-class support vector machine (OCSVM). The OCSVM requires only one example, or class of data (i.e., positive cases), which are used to maximize the space between these data and the origin in high-dimensional feature space [31,32]. In essence, this approach attempts to define and minimize a hypersphere wherein most of the data are found and thereby define a “typical” observation or gait cycle data set. This decision boundary can then be used as a classifier to determine if new data fits within this hypersphere (i.e., positive case or “typical” gait cycle) or outside of this hypersphere (i.e., negative case, “atypical,” or outlier gait cycle) [33]. This method has been shown to be successful in detecting outlier cases across a number of different machine learning applications [20,33,34,35]. In the current investigation, we used “fitcsvm,” available from Matlab (The MathWorks Inc., Natick, MA, USA), which utilizes the algorithm defined by Schölkopf et al. [31,32]. The boundary definition for this classifier was trained using the baseline features retained following the above-mentioned data reduction technique (i.e., PC scores depicting 95% of total variance). The training of this boundary was done using a Gaussian kernel function, in combination with a “ν” parameter for regularization. This parameter was chosen based on the value that achieved less than 1% outliers in a randomly selected 20% cross-validation set from baseline data. In other words, the OCSVM decision boundary was set wide enough to include 99% of the baseline gait cycles and thereby define a “typical” gait cycle data set. It should also be noted that to ensure the method was entirely subject-specific, this regularization parameter definition was conducted separately within each individual. Finally, post-intervention data were tested to determine the percentage of gait cycles that were defined as outliers given the baseline-derived multivariate boundary threshold. 

**Figure 2:**
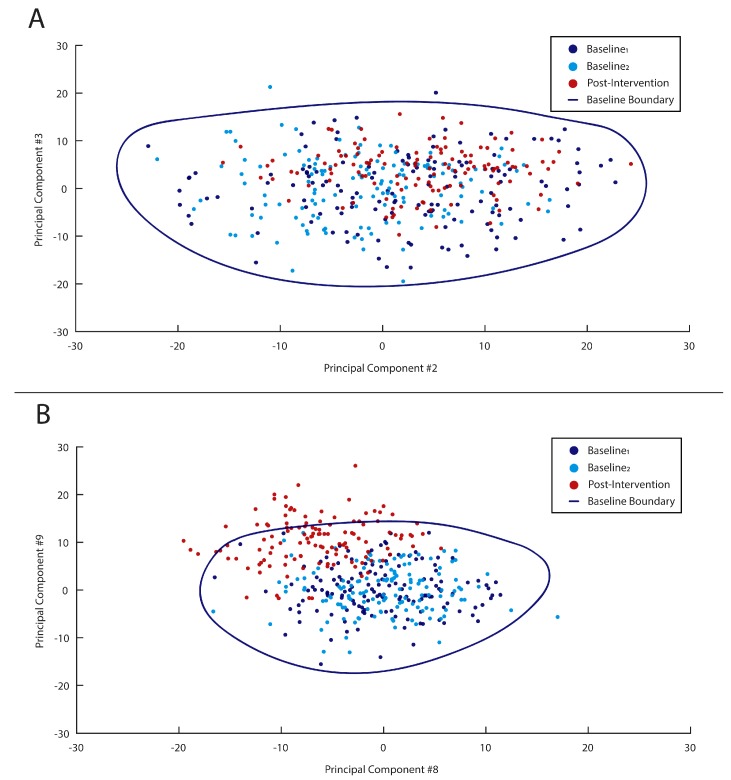
Simplified visualizations of boundary definitions (blue line) defined by baseline data (dark blue and light blue circles) and tested on post-intervention data (red circles). In example (**A**), no post-intervention gait cycles are defined as outliers, while in example (**B**) approximately 30% are viewed as outliers.

Two simplified visualizations of this boundary definition, using only 2 PCs, are shown in Figure 2. Figure 2A shows an example where no post-intervention gait cycles fell outside the baseline-defined OCSVM boundary (i.e., 0% outliers), suggesting no change in gait patterns following the intervention. Alternatively, in Figure 2B, a large number of post-intervention gait cycles fell outside the baseline-defined OCSVM boundary (i.e., approximately 30% outliers), suggesting the patient’s gait pattern has changed in response to the intervention. However, it should again be noted that the OCSVM boundary and subsequent results were based on a high-dimensional PC space for each subject. Alternatively, these examples in Figure 2 represent only a 2-dimensional sub-space for ease of visualization and understanding.

#### 2.3.4. Statistical Analysis

The primary variables of interest were: (i) percentage of gait cycles defined as outliers in the post-intervention gait trial; and (ii) average improvement in self-reported subscales of pain, symptoms, function in daily living and knee related quality of life (i.e., post-intervention scores—baseline scores). A non-parametric correlation, Spearman’s rank correlation (ρ), was used to assess the association between these two variables. A correlation of 0.10–0.29, 0.30–0.49 and 0.5+ were interpreted as small, medium and large, respectively [36]. Any subjects that reported no change or negative change were assessed as a zero-net change, based on the purpose of identifying the relationship of gait pattern deviation to clinical improvements. 

## 3. Results

**Figure 3:**
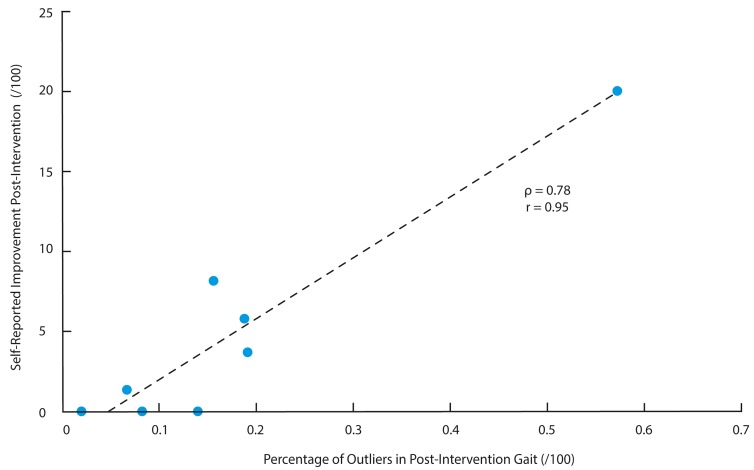
The percentage of outlier in post-intervention data displayed a large association with the self-reported improvement in post-intervention (i.e., change in Knee Injury Osteoarthritis Outcome Scores subscales): Spearman rank correlation (ρ) = 0.78 and Pearson correlation coefficient (r) = 0.95.

The average number of PCs retained to describe 95% of the total variance in each subject was 84 (5). The average percentage of gait cycles defined as outliers in the 20% cross-validated baseline data and post-intervention gait trial data were 0.5 (0.4)% and 17.7 (17.1)%, respectively. The best regularization parameters (ν) selected for the single-subject boundary thresholds were found to range between 0.1–0.8, with an average value of 0.4 (0.3). The percentage of outlier gait cycles in the post-intervention gait session achieved a large association (ρ = 0.78; *p* = 0.02) with the improvement in self-reported clinical outcomes following the intervention. This association, with non-parametric (Spearman rank correlation; ρ) and parametric coefficients (Pearson’s correlation; r), is visualized using a scatter plot in Figure 3.

## 4. Discussion

The purpose of this study was to establish a proof-of-concept for the use of a machine learning approach for assessing patient-specific changes in gait following an exercise therapy intervention. In support of our hypothesis, patients who benefited most from the exercise intervention also demonstrated the greatest overall change in gait patterns, as defined by the single-subject OCSVM models. This finding was demonstrated by the significant association (ρ = 0.78; *p* = 0.02) between the percentage of outlier gait cycles observed in post-intervention gait and clinical outcome improvement. In the context of previous univariate models examining the association of changes in muscle strength (r^2^ = 0.28–0.31; [37]) or pain sensitization (ρ = 0.28–0.35; [38]) to changes in self-reported outcomes in knee OA patients following exercise, the current association is comparatively large. To our knowledge, this is the first study to integrate pattern recognition algorithms with wearable technology to define objective, subject-specific biomechanical outcomes related to clinical improvements. Moreover, the current findings support the recent recommendation of utilizing subject-specific models in wearable sensor research [19].

In general, these findings are in accordance with a similar subject-specific one-class model approach used by Cola et al. [22], however there are a number of important distinctions. First, Cola et al. [22] examined artificially prescribed gait perturbations in healthy subjects to identify negative deviations from their “typical” gait patterns. In contrast, the current study introduced a 6-week exercise intervention to identify presumably positive deviations from a knee OA patient’s “typical” pattern. In addition to the contrasting clinical application, there remain a number of differences in the method itself, such as; (i) the number of sensors used (3 vs. 1), (ii) the number of days used to train baseline patterns (2 vs. 1) and (iii) the algorithm to define typical patterns (SVM vs. k-means). However, perhaps the most important distinction between the current study and that of Cola et al. [22] is the manner in which the features and threshold parameters were selected. Specifically, the work by Cole et al. [22] introduced gait perturbations in a controlled and standardized manner, which afforded the authors the ability to deliberately select the best features (i.e., 11 gait variables out of 43 total) and threshold parameters for identifying these perturbations in future gait data collections. While this a priori knowledge of the gait perturbations may be appropriate for identifying a known or consistent gait perturbation, it is not possible in clinical practice where the potential change is unknown. Alternatively, the current study uses a completely unsupervised approach, with no a priori feature selection or information of how a patient’s gait may or may not change. In doing so, we were able to define a holistic and objective measure of subject-specific changes in gait mechanics following an exercise intervention, something rarely seen in previous OA research.

While exercise interventions have consistently demonstrated improvements in the pain and function of knee OA patients, identifying concomitant changes in gait patterns has been rarely reported using conventional group-based methods [39,40,41,42]. Given that much of this research has examined single, discrete variables (e.g., knee adduction moment), the sensitivity of this type of univariate statistical approaches is questionable [17]. In contrast, the current study supports previous research suggesting examining multivariate and/or multi-segment changes may better quantify the overall biomechanical changes that occur after an exercise intervention [43,44]. Nevertheless, these multivariate changes often remain limited when assessed in conventional group-based analyses [44]. This further suggests that exercise interventions may not elicit any consistent change in gait patterns, univariate or multivariate, across heterogeneous diseases such as knee OA [39,40,41,42]. Therefore, a significant strength of the current study is the introduction of an alternative, single-subject model to track multivariate, multi-segment changes in gait biomechanics. Further, this approach is directly aligned with the ongoing shift towards precision medicine and personalized treatment approaches [45,46].

**Figure 4:**
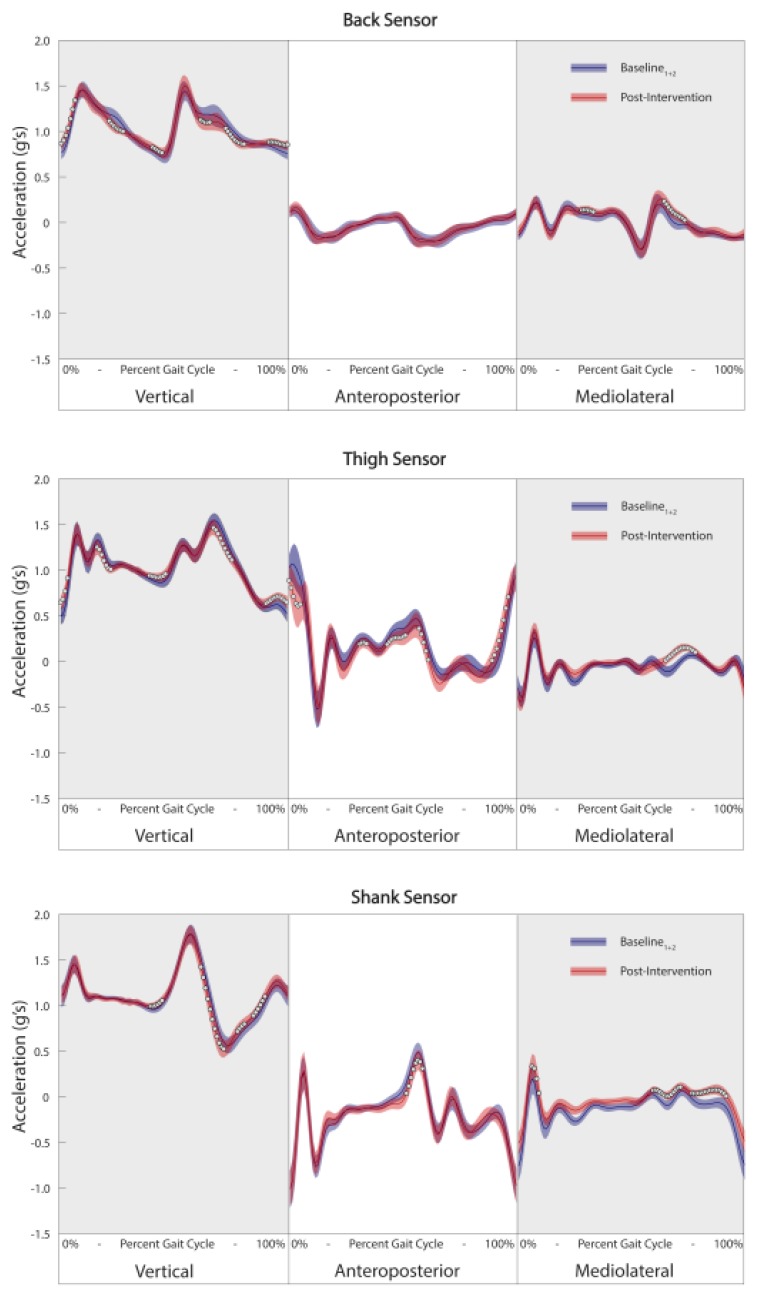
Example of waveform analysis of three-dimensional linear accelerations from the back (**top**), thigh (**middle**) and shank (**bottom**) visualizing changes in post-intervention data (red line) compared to baseline data (blue line). Data is presented from the patient who demonstrated the greatest number of gait cycle outliers post-intervention. White circles represent areas of the waveform where differences between baseline and post-intervention data are statistically significant (Holm-Bonferroni corrected *p*-value of less than 0.05) and have a large effect size (Cohen’s d > 0.8).

Given the complex nature of the proposed analysis, it becomes increasingly important to concentrate on ways to translate this information to the clinician and patient in a relevant and meaningful manner. The first and most simplistic way to translate this information is in the form of a percentage score from 0% to 100%, with 0% being no change in gait pattern and 100% being a complete change in overall gait pattern. In this instance, the output becomes easily interpretable but a somewhat black box assessment of the overall change. Alternatively, these holistic changes in gait patterns could be further stratified into sensors/segments and planes of movement to identify which sensors and axes were most important in driving the overall change, similar to Phinyomark et al. [47]. Finally, a more conventional waveform analysis could be used to examine the specific changes that may have occurred in relation to the baseline boundary. A brief representative example of this analysis is presented in Figure 4. In this example, while the thigh sensor appears to contain the most important changes for this individual, it is evident that there are a number of multi-segment changes occurring as well. Although many of these changes appear subtle, it remains unclear whether such changes accumulating over thousands of gait cycles per day may relate to a clinically important change in the mechanical loading of the knee joint. Therefore, the current study remains a proof-of-concept approach assessing this novel evidence-informed approach. Further work is required to develop highly specific and clinically relevant data visualizations and outcomes, as well as relating these biomechanical outcomes to long-term disease progression.

### Limitations and Future Directions

As is with any proof-of-concept study, there are a number of important limitations that must be discussed and addressed in future research. First, the sample size for this study was relatively small. Nevertheless, given the subject-specific model utilized the analysis, the limited sample size would only have affected strength of the relationship between gait cycle outliers and clinical improvements across the patients. However, it would not influence the fit of any individual models themselves and thus there is no increased risk of overfitting related to the sample size. To this point, additional subjects would be beneficial in filling out the space in Figure 3 but would not change the current position of any data points in the space. Second, two baseline trials, amassing a total of 5 min of walking data with an average of 270 gait cycles, were used to train each subject’s typical pattern. Given the highly controlled data collection environment, gait cycles collected over two separate days were thought to be sufficient in order to account for any potential between-day errors or variability. Nevertheless, future research should examine the impact of including more than 2 days of gait data as this will likely become more important in less controlled gait assessment protocols. Another potential limitation is the amount of data collected and utilized for the analysis (i.e., continuous waveform data from three sensors). While on-board preprocessing would likely provide the most efficient management of data, based on our previous research the limiting factor in this may be the event detection itself [48]. Nevertheless, this topic remains outside the scope of the current proof-of-concept study and future work may look to examine the most efficient ways preprocess and package the data for analysis. Lastly, our data was filtered with a 10 Hz low-pass filter as we were primarily interested in the overall pattern of gait, rather than high frequency impact accelerations or vibrations. Future research may look to include additional frequency-based parameters in their analysis to further define potential changes in gait.

## 5. Conclusions

The current study demonstrates a successful proof-of-concept for the use of a subject-specific one-class model for identifying individualized changes in gait patterns in response to an exercise intervention. The changes in gait patterns observed with this method were found to be associated with improvements in self-reported clinical measures, following the 6-week rehabilitation protocol. Therefore, this novel method effectively integrates machine learning and wearable technology to provide an objective and evidence-informed way to understand clinically important changes in human movement patterns.
